# APIS: an updated parentage assignment software managing triploids induced from diploid parents

**DOI:** 10.1093/g3journal/jkae143

**Published:** 2024-07-02

**Authors:** Julien Roche, Ronan Griot, François Allal, Mathieu Besson, Pierrick Haffray, Pierre Patrice, Florence Phocas, Marc Vandeputte

**Affiliations:** SYSAAF (French Poultry and Aquaculture Breeders Technical Centre), 35042 Rennes, France; Université Paris-Saclay, INRAE, AgroParisTech, GABI, 78350 Jouy-en-Josas, France; SYSAAF (French Poultry and Aquaculture Breeders Technical Centre), 35042 Rennes, France; Université Paris-Saclay, INRAE, AgroParisTech, GABI, 78350 Jouy-en-Josas, France; MARBEC, Univ Montpellier, CNRS, Ifremer, IRD, INRAE, 34250 Palavas-les-Flots, France; MARBEC, Univ Montpellier, CNRS, Ifremer, IRD, INRAE, 34250 Palavas-les-Flots, France; SYSAAF (French Poultry and Aquaculture Breeders Technical Centre), 35042 Rennes, France; SYSAAF (French Poultry and Aquaculture Breeders Technical Centre), 35042 Rennes, France; SYSAAF (French Poultry and Aquaculture Breeders Technical Centre), 35042 Rennes, France; Université Paris-Saclay, INRAE, AgroParisTech, GABI, 78350 Jouy-en-Josas, France; MARBEC, Univ Montpellier, CNRS, Ifremer, IRD, INRAE, 34250 Palavas-les-Flots, France

**Keywords:** parentage assignment, triploids, SNP, R software, shiny interface

## Abstract

In aquaculture, sterile triploids are commonly used for production as sterility gives them potential gains in growth, yields, and quality. However, they cannot be reproduced, and DNA parentage assignment to their diploid or tetraploid parents is required to estimate breeding values for triploid phenotypes. No publicly available software has the ability to assign triploids to their parents. Here, we updated the R package APIS to support triploids induced from diploid parents. First, we created new exclusion and likelihood tables that account for the double allelic contribution of the dam and the recombination that can occur during female meiosis. As the effective recombination rate of each marker with the centromere is usually unknown, we set it at 0.5 and found that this value maximizes the assignment rate even for markers with high or low recombination rates. The number of markers needed for a high true assignment rate did not strongly depend on the proportion of missing parental genotypes. The assignment power was however affected by the quality of the markers (minor allele frequency, call rate). Altogether, 96–192 SNPs were required to have a high parentage assignment rate in a real rainbow trout dataset of 1,232 triploid progenies from 288 parents. The likelihood approach was more efficient than exclusion when the power of the marker set was limiting. When more markers were used, exclusion was more advantageous, with sensitivity reaching unity, very low false discovery rate (<0.01), and excellent specificity (0.96–0.99). Thus, APIS provides an efficient solution to assign triploids to their diploid parents.

## Introduction

Triploids, which are individuals bearing 3 sets of chromosomes instead of 2, are commonly used in plant, fish, and shellfish breeding (Piferrer *et al*. 2009; Wang *et al*. 2016). The benefits of using triploids are diverse and can imply higher growth due to larger cell size at least in plants (Sugiyama 2005), although this effect does not seem to be general (Tsukaya 2013) especially in fish. One of the main interests of triploids is that they are most of the time sterile, which has several implications, (1) a higher growth, mainly after the age at maturity, due to a lack of investment in gonads and reproduction (Quillet *et al*. 1988), (2) a higher organoleptic quality due to the absence of mature gonads (oyster), seeds (citrus), or to the sparing of lipid and pigments, not transferred from the edible muscle to the gonads (trout), and (3) a much lower risk of genetic introgression of farmed genotypes into wild populations (Benfey 2016).

As triploids have benefits for farming, as any farmed genotype, they require genetic improvement, which implies in most cases the establishment of pedigrees. When families and/or genotypes are grown altogether in a common plot or tank to avoid confounding genetic and environmental effects, it is necessary to use genetic markers to assign the tested genotypes to their parents (see Vandeputte and Haffray 2014 for the case of cultured fish).

Triploid induction in fish is performed by applying temperature or pressure treatments to fertilized eggs in order to prevent the extrusion of the second polar body, i.e. by suppressing telophase II of the meiosis (Piferrer *et al*. 2009). Such triploids then have 2 sets of chromosomes from the maternal genome and 1 from the paternal genome.

Triploid trout were a major improvement for the aquaculture industry. Indeed, triploid individuals have better performance than diploids for filet yield and growth, especially when the production cycle exceeds 2 years, thus boosting the production of large rainbow trout for the smoked fillet market (Chourrout *et al*. 1986; Piferrer *et al*. 2009). For Atlantic salmon which are mostly reared in cages, the production of sterile fish is essentially seen as a way to decrease the potential genetic contamination of wild populations (Galbreath *et al*. 1994; Glover *et al*. 2013; Benfey 2016).

While the first family-based breeding programs for fish used separate rearing of families to establish pedigrees, now in many fish breeding programs, fish are reared in batches of full and half-sib families mixed in a common tank before individual identification is possible (Haffray *et al*. 2018). This helps reduce the confusion of genetic and environment effects. However, in such programs, pedigree information remains unknown until it can be recovered *a posteriori* by using genetic markers for parentage assignment (Vandeputte and Haffray 2014). In typical fish breeding programs, only diploid fish are assigned to their parents and evaluated for their breeding value. The capacity to perform genetic improvement of the final triploid sterile commercial product is however dependent on the genetic correlation between diploid and triploids traits. Although little literature is available on the subject, the heritability of traits may differ between diploid and triploid progenies (Johnson *et al*. 2007), and there may be re-ranking of families when performance of diploids and triploids are compared (Blanc *et al*. 2001; Bonnet *et al*. 1999, 2002). In addition, the genetic models applied to evaluate breeding values of diploids and triploids differ from each other (Hamilton and Kerr 2018). In any case, to maximize genetic gains in triploids, it may then be necessary to evaluate breeding values on triploid siblings of the selection candidates, which implies the need to recover their pedigree.

Although methodologies for parentage assignment of triploids to their diploid parents have been previously proposed (Miller *et al*. 2016; Grashei *et al*. 2020), none are to date implemented in a readily usable software. In addition, while the method of Grashei *et al*. (2020) applies to induced triploids in fish, the method of Miller *et al*. (2016) only applies to triploids produced from the mating of a diploid and a tetraploid parent, as is the case in oysters.

In this paper, we present how we adapted the parentage assignment R package APIS (Griot *et al*. 2020) to handle triploid individuals from diploid parents, obtained by second polar body retention after fertilization (which is the typical case in finfish—and widely applied in salmonids). We use both likelihood and exclusion methodologies, and compare their efficiency in different cases, both with real and simulated data.

## Material and methods

### Adaptation of APIS for parentage assignment of triploid individuals

The R package APIS (Griot *et al*. 2020) was initially designed to assign diploid individuals to their diploid parents genotyped with co-dominant genomic markers (usually SNPs or microsatellites) using primarily the distribution of the Mendelian transmission probability observed in the offspring to assign. The Mendelian transmission probability of a parent pair to an offspring (i.e. the probability of the offspring genotype, conditional to the genotype of the potential parents studied) was computed in tables that give the likelihood of the offspring genotype at 1 marker, knowing the genotypes of the sire and the dam at this marker.

To adapt APIS to triploid individuals, we created 3 new likelihood tables (Tables 1–3), 1 for each possible triploid offspring genotype (AAA, ABB, ABC). To perform parentage assignment by exclusion, similar tables were produced, which reveal incompatibilities between offspring and parental genotypes (Tables 4–6). Tables for diploid offspring remain the same as in the first version of APIS (Griot *et al*. 2020).

**Table 1.** Likelihood table for a marker with a homozygous (AAA) triploid offspring, showing the probability of the offspring genotype conditional on the parental genotypes.

Sire\dam	AA	AB	BB	Missing
AA	1	0.5 ∗ (1 − *r*)	*e*	*r* ∗ fA^2^ + (1 − *r*) ∗ fA
AB	0.5	0.25 ∗ (1 − *r*)	*e*	(*r* ∗ fA^2^ + (1 − *r*)fA) ∗ 0.5
BB	*e*	*e*	*e*	*e*
Missing	fA	0.5 ∗ (1 − *r*) ∗ fA	*e*	*r* ∗ fA^3^ + (1 − *r*) ∗ fA^2^

B = any allele that is not A; fA, fB = frequencies of alleles A and B in the offspring population analyzed; *e* = arbitrary value (here fixed to 0.01) for genotyping error; *r* = recombination rate between the centromere and the marker.

**Table 2.** Likelihood table for a marker with a heterozygous (ABB) triploid offspring, showing the probability of the offspring genotype conditional on the parental genotypes.

Sire\dam	AA	AB	BB	AC	BC	CC	Missing
AA	*e*	0.5 ∗ (1 − *r*)	1	*e*	0.5 ∗ (1 − *r*)	*e*	(1 − *r*) ∗ fAfB + fB^2^ + (1 − *r*) ∗ fBfC
AB	*e*	0.5–0.25 ∗ (1 − *r*)	0,5	*e*	0.25 ∗ (1 − *r*)	*e*	(1–0.5 ∗ (1 − *r*)) ∗ fAfB + 0.5 ∗ fB^2^ + 0.5 ∗ (1 − *r*) ∗ fBfC
BB	*e*	*r*	*e*	*e*	*e*	*e*	2fAfB ∗ *r*
AC	*e*	0.25 ∗ (1 − *r*)	0,5	*e*	0.25 ∗ (1 − *r*)	*e*	0.5 ∗ (1 − *r*) ∗ fAfB + 0.5 ∗ (1 − *r*) ∗ fBfC + 0.5 ∗ fB^2^
BC	*e*	0.5 ∗ *r*	*e*	*e*	*e*	*e*	fAfB ∗ *r*
CC	*e*	*e*	*e*	*e*	*e*	*e*	*e*
Missing	*e*	0.5 ∗ (1 − *r*) ∗ fA + *r* ∗ fB	fA	*e*	0.5 ∗ (1 − *r*) ∗ fA	*e*	fA ∗ ((1 − *r*) ∗ fAfB + fB^2^ + (1 − *r*) ∗ fBfC) + fB^2^fA ∗ 2 ∗ *r*

C = any allele that is not A or B; fA, fB, fC = frequencies of alleles A, B, and C in the offspring population analyzed; *e* = arbitrary value (here fixed to 0.01) for genotyping error; *r* = recombination rate between the centromere and the marker.

**Table 3.** Likelihood table for a marker with a heterozygous (ABC) triploid offspring, showing the probability of the offspring genotype conditional on the parental genotypes.

Sire\dam	AA	AB	BB	AC	BC	CC	AD	BD	CD	DD	Missing
AA	*e*	*e*	*e*	*e*	(1 − *r*)	*e*	*e*	*e*	*e*	*e*	2fBfC ∗ *r*
AB	*e*	*e*	*e*	0.5 ∗ *r*	0.5 ∗ *r*	*e*	*e*	*e*	*e*	*e*	fBfC ∗ *r* + fAfC ∗ *r*
BB	*e*	*e*	*e*	*r*	*e*	*e*	*e*	*e*	*e*	*e*	2fAfC ∗ *r*
AC	*e*	0.5 ∗ *r*	*e*	*e*	0.5 ∗ *r*	*e*	*e*	*e*	*e*	*e*	fAfB ∗ *r* + fBfC ∗ *r*
BC	*e*	0.5 ∗ *r*	*e*	0.5 ∗ *r*	*e*	*e*	*e*	*e*	*e*	*e*	fAfB ∗ *r* + fAfC ∗ *r*
CC	*e*	*r*	*e*	*e*	*e*	*e*	*e*	*e*	*e*	*e*	2fAfB ∗ *r*
AD	*e*	*e*	*e*	*e*	0.5 ∗ *r*	*e*	*e*	*e*	*e*	*e*	fBfC ∗ *r*
BD	*e*	*e*	*e*	0.5 ∗ *r*	*e*	*e*	*e*	*e*	*e*	*e*	fAfC ∗ *r*
CD	*e*	0.5 ∗ *r*	*e*	*e*	*e*	*e*	*e*	*e*	*e*	*e*	fAfB ∗ *r*
DD	*e*	*e*	*e*	*e*	*e*	*e*	*e*	*e*	*e*	*e*	*e*
Missing	*e*	*r* ∗ fC	*e*	*r* ∗ fB	*r* ∗ fA	*e*	*e*	*e*	*e*	*e*	*r* ∗ 6 ∗ fAfBfC

D = any allele that is not A, B, or C; fA, fB, fC = frequencies of alleles A, B, and C in the offspring population analyzed; *e* = arbitrary value (here fixed to 0.01) for genotyping error; *r* = recombination rate between the centromere and the marker.

**Table 4.** Exclusion table for a marker with a homozygous (AAA) triploid offspring, showing whether offspring genotype is incompatible with 1 parental genotype or with the combination of the parental genotypes (1) or is incompatible with any of the parental genotypes (2).

Sire\dam	AA	AB	BB	Missing
AA	0	0	1	0
AB	0	0	1	0
BB	1	1	2	1
Missing	0	0	1	0

B = any allele that is not A.

**Table 5.** Exclusion table for a marker with a heterozygous (ABB) triploid offspring, showing whether offspring genotype is incompatible with 1 parental genotype or with the combination of the parental genotypes (1) or is incompatible with any of the parental genotypes (2).

Sire\dam	AA	AB	BB	AC	BC	CC	Missing
AA	1	0	0	1	0	1	0
AB	1	0	0	1	0	1	0
BB	1	0	1	1	1	1	0
AC	1	0	0	1	0	1	0
BC	1	0	1	1	1	1	0
CC	2	1	1	2	1	2	1
Missing	1	0	0	1	0	1	0

C = any allele that is not A or B.

**Table 6.** Exclusion table for a marker with a heterozygous (ABC) triploid offspring, showing whether offspring genotype is incompatible with 1 parental genotype or with the combination of the parental genotypes (1) or is incompatible with any of the parental genotypes (2).

Sire\dam	AA	AB	BB	AC	BC	CC	AD	BD	CD	DD	Missing
AA	1	1	1	1	0	1	1	1	1	1	0
AB	1	1	1	0	0	1	1	1	1	1	0
BB	1	1	1	0	1	1	1	1	1	1	0
AC	1	0	1	1	0	1	1	1	1	1	0
BC	1	0	1	0	1	1	1	1	1	1	0
CC	1	0	1	1	1	1	1	1	1	1	0
AD	1	1	1	1	0	1	1	1	1	1	0
BD	1	1	1	0	1	1	1	1	1	1	0
CD	1	0	1	1	1	1	1	1	1	1	0
DD	2	1	2	1	1	2	2	2	2	2	1
Missing	1	0	1	0	0	1	1	1	1	1	0

D = any allele that is not A, B, or C.

One specificity of triploid individuals produced by blocking the extrusion of the second polar body from the eggs after fertilization is that possible genotypes depend on recombination events that occur during the first meiotic division in the female gametes. As the second polar body is retained, each triploid offspring carries 2 chromosomes of maternal origin. Thus, while a female which is homozygous (AA) at a marker will only produce AA gametes, a heterozygous (AB) female will produce either AA and BB gametes in equal proportions when there is no recombination event between the centromere and the marker, or only AB gametes in the case of a recombination event. The recombination rate ranges from 0 when the marker is located on the centromere to 1 if the marker is at the extremity of the chromosome arm, in case of full interference. Full interference happens when there is 1 and only 1 crossing over per chromosome arm, which seems to be close to reality at least in rainbow trout (Guyomard *et al*. 2006). However, as the distance to the centromere of each marker is not expected to be precisely known in most farmed fishes, we set a default value for this parameter to an average of 0.5 in APIS, an assumption that will be tested for its validity.

As mentioned in Griot *et al*. (2020), the value of the Mendelian transmission probability when the genotype of the tested parent pair is incompatible with the offspring genotype at the marker was set to *e* = 0.01 to account for genotyping error, as first suggested by Sancristobal and Chevalet (1997). As highlighted by Boichard *et al*. (2014), the true genotyping error may vary among methods and species, but the value of *e* is not critical for parentage assignment, as soon as it is small enough to penalize incompatibilities, but different from zero to avoid exclusion based on a single incompatibility. In the diploid version of APIS, we showed that using *e* = 0.01 for likelihood estimation could perfectly manage genotyping errors even when the true error rate was as high as 3% (Griot *et al*. 2020).

Beyond the management of triploids, new functions were introduced in APIS, including an automated determination of number of mismatches authorized for parentage assignment by exclusion, and a Shiny app to facilitate the use of APIS for users not familiar with R software. Those are implemented both for diploid and triploid offspring. Details are given in File 1.

The APIS package including these new functions is available on the CRAN repository at https://cran.r-project.org/package=APIS.

### Comparison with the method of Grashei *et al.* (2020)

As indicated in the introduction, the exclusion-based method proposed by Grashei *et al*. (2020) is applicable in our case, however it has not been implemented in a publicly usable software. We coded it in Excel-VBA (File 2) and tested it on 2 cases representative of real situations in aquaculture: we simulated 1,000 offspring from a full factorial mating of 100 sires and 100 dams, to be assigned with 100 or 200 SNP markers of MAF 0.5, with a marker-centromere recombination rate of 0.5 and a genotyping error rate of 1%. This was repeated 5 times for 100 markers and 5 times for 200 markers, and the same simulated datasets were processed with APIS using the exclusion method and default parameters.

### Validation of triploid assignment rates

Rainbow trout genotype data were obtained for 1,232 triploid rainbow trout offspring induced by pressure treatment and their 288 diploid parents from “Les Aquaculteurs Bretons” (Plouigneau, France) selective breeding program (Vandeputte *et al*. 2019). The 98 sires and 190 dams had been crossed in a partial factorial design, with 10 distinct full factorial crosses with 8–10 sires and 17–24 dams each, producing a theoretical number of 1,862 full-sib families. All those individuals were genotyped for 57,501 SNP markers using the Rainbow Trout Axiom 57K SNP array from ThermoFisher (Palti *et al*. 2015). Genotype calling for triploid offspring was performed with the R package GenoTriplo (Roche *et al*. 2024) , while genotyping of the diploid parents was performed using the Axiom Analysis Suite from ThermoFisher. In the end, 38,033 high-quality markers were kept after quality control following D’Ambrosio *et al*. (2019). To get a reference pedigree, we used APIS on the best 1,000 and 2,000 markers (based on marker call rate and MAF maximization) and checked that (1) 100% of the offspring were assigned to a single parental pair, (2) there were no differences in pedigree between the results with 1,000 and 2,000 markers, showing that 1,000 markers provided the best assignment possible with our dataset, and (3) both parents of each offspring came the same recorded factorial mating. The number of full-sib families effectively produced with the partial factorial mating system used (715) was only 3.8% of the total number of the potential 18,620 families that could have been produced from 98 sires and 190 dams. Thus, an incorrect parental pair would likely be outside of the recorded mating plan in 96.2% of the cases, which was never the case. With these 3 independent controls, we were confident the pedigree obtained with the best 1,000 markers was the true pedigree.

From the real rainbow trout dataset, we also simulated offspring by mating *in silico* randomly chosen sires and dams from the whole potential parent dataset. Each parental marker was inherited following the Mendelian rules. Each sire and dam transmitted 1 of its 2 alleles with a probability of 0.5, while the dam, due to the induced triploidy, also had to transmit a second allele to the offspring. The probability for the dam to give twice the allele of the same chromosome was given by the recombination rate which was set at 0.5 (to match the default recombination rate of the procedure). Genotyping errors were simulated by randomly sampling an allele from A or B. The genotyping error rate was set at 1%, a relatively high value for SNPs, for which error rates are more in the 0.1–0.6% range when using genotyping arrays or high coverage next generation sequencing data (Jiang *et al*. 2013; Kvale *et al*. 2015; Wall *et al*. 2014). This rather high value was chosen to ensure the robustness of our parentage assignment method.

### Effect of the default value for recombination rate

The recombination rate is a key parameter used in the likelihood tables (Tables 1, 2 and 3). To test the effect of the recombination occurring during meiosis on the efficiency of parentage assignment, 3 populations of 500 simulated individuals with 100 randomly chosen markers were simulated. Each was simulated with a different recombination rate: 0.25, 0.5 and 0.75. Then, we assigned those simulated populations while varying the APIS procedure's general recombination rate (similar for all markers in a given assignment run) from 0.05 to 0.95 with a step of 0.05, to evaluate the effect of the recombination rate parameter on the assignment rate with the likelihood method. Each simulation was replicated 10 times.

### Evaluation of the assignment rates

Three complementary approaches were tested to evaluate assignment rates with APIS, (1) “real random” using the real reference rainbow trout dataset, with randomly chosen markers (2) “real best” using the same dataset but selecting the “best” markers for call rate and MAF and (3) “simulated random” using genotypes of the rainbow trout parents to simulate offspring *in silico* and assigning them using randomly chosen markers.

The first 2 approaches made the hypothesis that the true parents were the ones identified by APIS using 1,000 markers (see before), while in the third one, the true parental pairs were known with certainty as offspring were simulated. In terms of genotyping errors, the first 2 approaches included real genotyping errors, while the third used simulated genotyping errors.

In each of the 3 approaches we subset 16, 32, 48, 96, 192, 384, 500, and 1,000 markers, and randomly sampled 90% (171 dams and 88 sires) and 50% (95 dams and 49 sires) of the parents to test the impact of 10 or 50% of missing parental genotypes on assignment rate. Each sampling of parents was replicated 10 times. Thus, we tested all the combined scenarios with 16, 32, 48, 96, 192, 384, 500, and 1,000 markers and 100%, 90%, and 50% of the parents having genotypes. Each assignment was done with a tolerated assignment error rate fixed at 5%.

In the “real random” approach, marker subsets were made at random and replicated 10 times. In the “real best” approach, the markers were sampled only once, as we selected the best of them (sorted by marker call rate and MAF in the offspring population—See Supplementary Table 1 in File 1).

Finally, for the “simulated random” approach, we simulated offspring by mating *in silico* randomly chosen sires and dams with a 1% genotyping error rate, as described previously.

To evaluate assignment efficiency, we evaluated 3 metrics:


$$\begin{array}{rl} \mathrm{Sensitivity} & =\;\frac{\mathrm{true}\;\mathrm{positives}}{\mathrm{positives}\;}\\ & =\frac{\mathrm{offspring}\;\mathrm{assigned}\;\mathrm{to}\;\mathrm{their}\;\mathrm{true}\;\mathrm{parents}}{\mathrm{offspring}\;\mathrm{with}\;\mathrm{both}\;\mathrm{parents}\;\mathrm{genotyped}}. \end{array}$$

Ideally, in a parentage assignment procedure, sensitivity should be close to 1. When this was not the case, we also estimated false discovery rate (FDR):


$$\mathrm{FDR}=\;\frac{\mathrm{false}\;\mathrm{positives}}{\mathrm{true}\;\mathrm{positives}+\mathrm{false}\;\mathrm{positives}}.$$

FDR quantifies errors in the pedigree returned by the software, and is thus a key parameter for the usability of this pedigree, as pedigree errors are known to lead to reduced selection response in breeding programs (Banos *et al*. 2001; Visscher *et al*. 2002) and to inappropriate genetic management of populations (Oliehoek and Bijma 2009).

Finally, in cases where not all offspring had both parents genotyped (and thus those offspring with missing parents were considered negatives), we also evaluated specificity:


$$\mathrm{Specificity}=\;\frac{\mathrm{true}\;\mathrm{negatives}}{\mathrm{negatives}}.$$

In parentage assignment applications, specificity is especially interesting in restocking of natural populations, where there is key interest in identifying individuals that are not the result of restocking (from known parents) but of natural matings from unknown parents (see e.g. Vandeputte *et al*. 2021).

## Results

### Comparison with the method of Grashei *et al.*

When using 100 SNP markers with MAF = 0.5 on a simulated dataset of 1,000 offspring with a marker-centromere recombination rate of 0.5 and a genotyping error rate of 1%, the method of Grashei *et al*. (2020) failed to assign any offspring in any of the 5 replicate trials. On the same trials, APIS run with default parameters and method “exclusion” assigned all 5,000 offspring to their true parental pair, reaching a sensitivity of 100.0%. With the same mating plan, using 200 markers, the sensitivity of the method of Grashei was 99.7%, while that of APIS was still 100.0%. FDR was zero for both software, as the failed assignments of the Grashei method were cases where the dam was correctly identified but the sire was unassigned.

### Effect of recombination rate

**Fig. 1.**
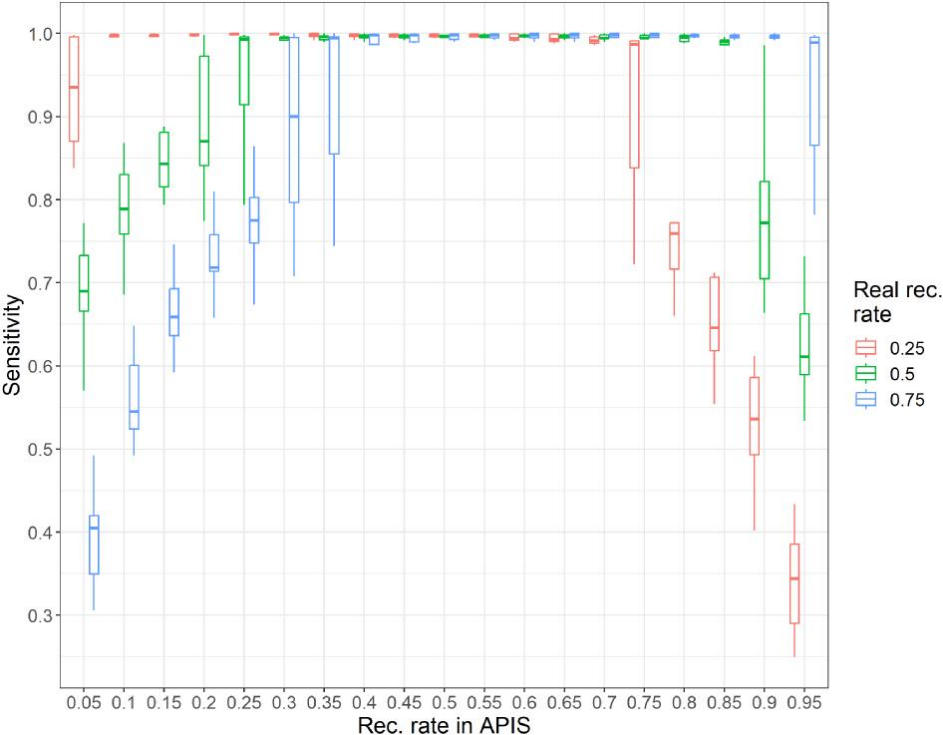
Sensitivity of parentage assignment with the likelihood method in APIS with 100 SNP markers as a function of the recombination rate parameter in APIS for populations with a simulated recombination rate of 0.25, 0.50, and 0.75 (10 replicates each).

The effect of recombination rate was tested on 500 offspring with 100 SNP markers with true recombination rates of 0.25, 0.50, and 0.75 while varying the internal recombination rate parameter of APIS from 0.05 to 0.95 (Fig. 1). The sensitivity reached 1.0 (all offspring correctly assigned) for all values of the internal recombination rate parameter of APIS ranging from 0.40 to 0.70, whatever the real recombination rate. The ranges of the parameter for which sensitivity reached 1.0 were larger for each value of the real recombination rate (from 0.10 to 0.65 for 0.25, from 0.20 to 0.70 for 0.50, and from 0.40 to 0.90 for 0 0.75).

When the method was set to “exclusion”, there was no impact of the recombination rate as the algorithm only counts what is not possible and takes recombination as a possible event in any case. The performance of exclusion was lower than that of likelihood, with a sensitivity of 0.89 only.

### Assignment rates in simulated and real datasets

Assignment rates were assessed using both the simulated and the real datasets with the likelihood and the exclusion methods.

**Fig. 2.**
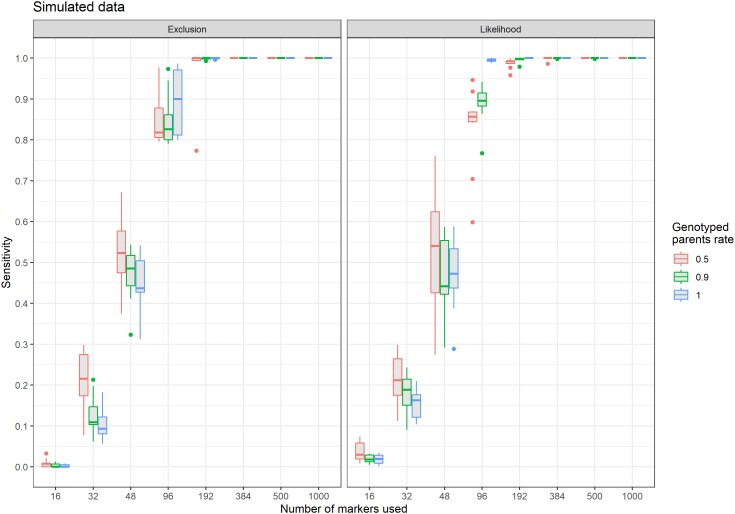
Sensitivity of APIS parentage assignment for triploid rainbow trout simulated offspring as a function of the number of markers used for populations with 50%, 90%, and 100% of the parents genotyped with the exclusion (left) and likelihood (right) methods (10 replicates).

With the simulated dataset, the amount of missing parents did not impact sensitivity with the exclusion method (Fig. 2). For the likelihood method, there was a real difference between missing parents modalities when 96 markers were used (Fig. 2): when all parents were genotyped, the average sensitivity exceeded 0.995 whereas it was lower (0.887 and 0.830) for the modalities with 90 and 50% of the parents genotyped, respectively. The average sensitivity exceeded 0.97 with 192 markers for both exclusion and likelihood, and was 0.999 or more as of 384 markers. This was true for all levels of missing parents.

**Fig. 3.**
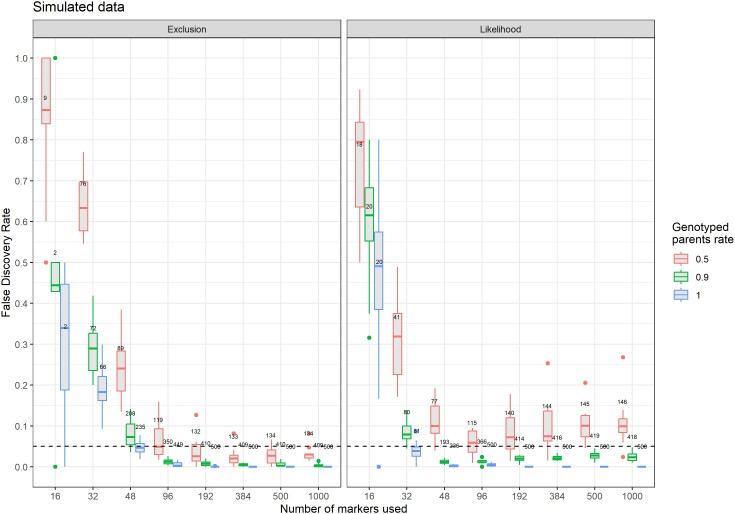
False discovery rate with APIS for triploid rainbow trout simulated offspring as a function of the number of markers used for populations with 50%, 90%, and 100% of the parents genotyped with the exclusion (left) and likelihood (right) methods (10 iterations). Values on top of boxplots are the mean number of assigned offspring out of the 500 simulated for each replicate. The dashed line represents the user-chosen FDR of 0.05.

Even though the algorithm did not provide high sensitivity values when used with less than 96 markers, it did not often wrongly assign parents to an offspring. When exclusion was used, the FDR decreased with the number of markers (Fig. 3). With 16 or 32 markers, FDR was high (>0.20) but the number of assigned offspring was really low (around 75 offspring with 32 markers, representing 15% of the total number of offspring). As of 96 markers, FDR was better controlled and its average value was lower than the user-chosen threshold of 0.05, although some replicates exceeded this value. Missing parental genotypes led to only a slight increase in FDR which however stayed below 0.05 in most cases. With the likelihood method, the FDR was lower than with exclusion for 32 and 48 markers. However, while it remained below the user-chosen threshold of 0.05 for cases with 0 and 10% of parents with missing genotypes, it surpassed it repeatedly (up to 0.10 on average), even with a high number of markers. When 50% of parents had missing genotypes, specificity was 0.98 or more as of 96 markers with exclusion, and 0.95 or more with likelihood. However, when only 10% of the parents had missing genotypes, with 96 markers or more, the performance was lower with a specificity higher than 0.96 with exclusion but ranging from 0.87 to 0.95 with likelihood.

**Fig. 4.**
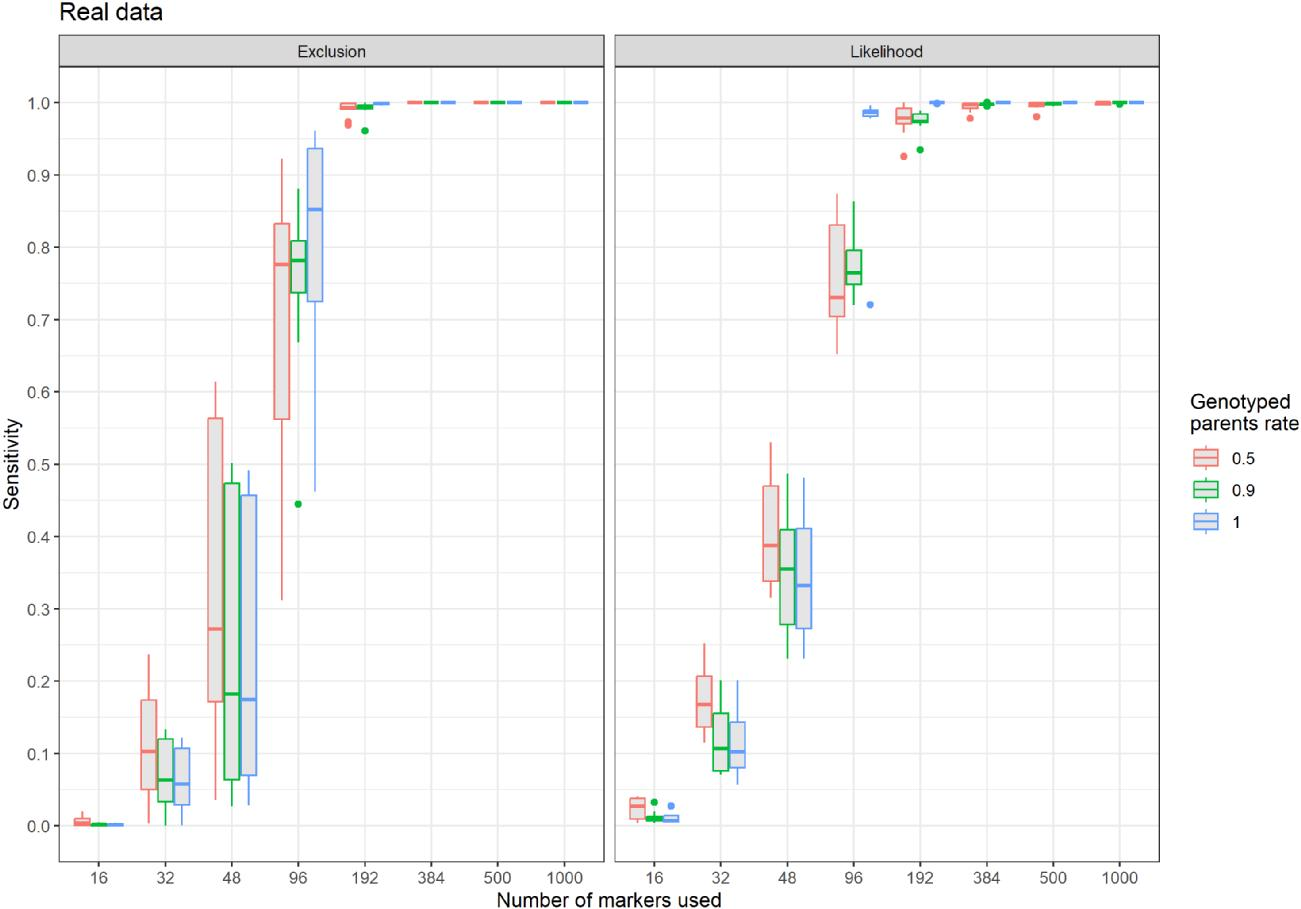
Sensitivity of assignment as a function of the number of SNP markers used for 1,232 rainbow trout triploid offspring populations with 50%, 90%, and 100% of the parents genotyped with the exclusion (left) and likelihood (right) methods (10 iterations). The “true” parental pair is the one obtained by exclusion using the 1,000 best markers and all parental genotypes.

When using the real rainbow trout dataset with randomly chosen markers, results were very similar and confirmed that the algorithm also works on a real dataset of triploid offspring genotypes. Taken as a whole, the results show that the likelihood approach had a better sensitivity (Fig. 4) when the power of the marker set was limiting (i.e. 48 and 96 markers) but reached unity only with 384 markers and more, when all parents are genotyped. With 384 markers and more, the sensitivity of exclusion was unity even with the highest number of parents with missing genotypes.

**Fig. 5.**
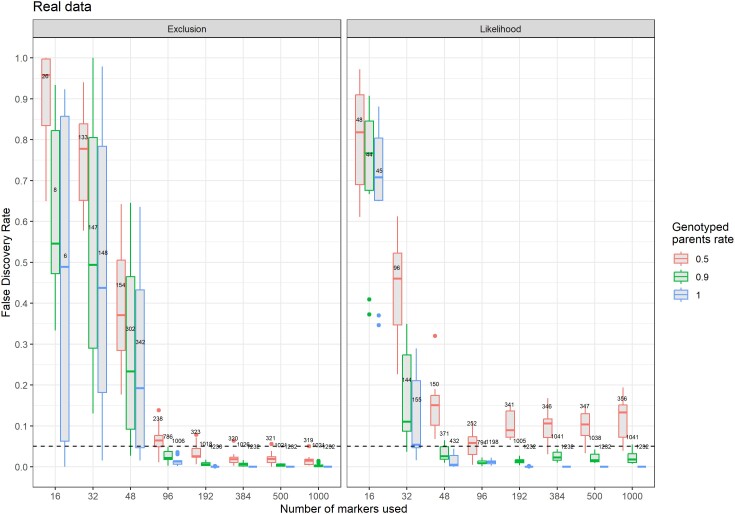
False discovery rate of APIS as a function of the number of SNP markers used for 1,232 rainbow trout triploid offspring with 50%, 90%, and 100% of the parents genotyped with the exclusion (left) and likelihood (right) methods. Values on top of boxplots are the mean number of assigned offspring (10 iterations). The dashed line represents the user-chosen 0.05 limit for FDR.

When all parents were genotyped, the FDR fell below the user-set value of 0.05 with 96 markers or more with exclusion, while only 48 markers were sufficient with likelihood (Fig. 5). With 192 markers or more, it was very close to zero for both methods. With 90% of the parents genotyped, the pattern was similar, but while FDR stayed below 0.05, it did not tend to 0 with the likelihood method whereas it reached 0 with the exclusion method when considering a large set of markers. When 50% of the parents were missing, the FDR with likelihood was higher than the user-set threshold with an average of 0.10, while it was below the threshold for most replicates when using exclusion. Specificity followed the same pattern observed with the simulated datasets.

### Impact of marker quality

The previous test, using randomly chosen markers, showed that exclusion was slightly less efficient in terms of sensitivity compared to likelihood, especially when there were no missing parental genotypes, and required around 192 markers to give good results. With the “best” markers (in terms of call rate and MAF, see Supplementary Table 1 in File 1), 96 markers were enough to have a sensitivity close to unity, gaining about 0.1 of sensitivity relative to that obtained with randomly chosen markers (Supplementary Fig. 1 in File 1). Even with only 50% of the parents genotyped, the sensitivity was 0.99 with 96 markers. With only 48 markers, the sensitivity exceeded 0.8 (respectively 0.86, 0.82, and 0.82 for 50%, 90%, and all parents genotyped). With the likelihood method, the impact of the quality of the markers on sensitivity was rather similar. With 96 markers, there was a 0.1 gain in sensitivity (except with all parents genotyped as sensitivity, in this case, was already close to 1 with randomly chosen markers) leading respectively to 0.81, 0.95, and 0.99 for 50%, 90%, and all parents genotyped. Thus selecting the best markers led to a real increase in sensitivity for the same number of markers.

The FDR was also impacted by the quality of the markers with the exclusion method. It was reduced by around 0.26 with the best 48 markers reaching respectively 0.12, 0.01, and 0.001 when 50%, 90%, and all parents were genotyped (Supplementary Fig. 1 in File 1). Even with 96 markers, there still was a small decrease of FDR although the FDR was already pretty close to 0 with random markers. The impact of marker quality on FDR was much lesser with the likelihood method. There was less than 0.1 reduction in the FDR when there were more than 32 markers. The reduction was close to zero when all or 90% of the parents were genotyped but, in this case, the FDR was already close to 0 with randomly chosen markers. A real reduction of FDR was observed when only 50% of the parents were genotyped, making the FDR fall below the user-set threshold of 0.05.

Specificity, when 96 markers or more were used, was similar to that observed with randomly selected markers (either in the real or in the simulated datasets) with a higher value when using exclusion (>0.99 for 50% parents genotyped, >0.96 for 90% parents genotyped) than with likelihood (≈0.99 for 50% parents genotyped, ≈0.90 for 90% parents genotyped).

## Discussion

The procedure implemented in APIS to assign triploid offspring to their diploid parents was accurate and can be used not only in rainbow trout but also in any fish breeding program to *a posteriori* retrieve the pedigree of induced triploid offspring. To our knowledge, APIS is the only publicly available parentage assignment software that can handle triploid individuals with 2 copies of the maternal genome and 1 of the paternal genome, which happens when triploid is induced by retention of the second polar body. Grashei *et al*. (2020) proposed an exclusion method to assign triploid individuals to their diploid parents, without implementing it in a freely available software. Their method differs from our exclusion method by 2 points:

1. They do not assign offspring to parental pairs but to parents, and retain the 2 most likely parents as the true parental pair, provided the proportion of exclusions is lower than the lowest value for the third most likely parent. Excluding sires and dams without taking into account the genotype of the other parent necessarily results in a lower exclusion power (Dodds *et al*. 1996); and
2. They do not use prior information on the sex of the parents, but deduce it from genotyping results. This necessarily results in lower performance as less information is used. For example, an ABB offspring in our case cannot be the offspring of a AA dam, but in their method these “dam-specific exclusions” are not used to exclude parents but to identify dams.

The Grashei method failed to assign any offspring in simulated cases where 100 SNP markers were used to assign 1,000 offspring from 100 sires and 100 dams, when APIS assigned 100% of the offspring to their correct parent pair. The reason for this very low efficiency is that when a limited number of markers is available, the likelihood that the minimum number of exclusions (based on opposed homozygotes) observed for the third best parent is zero is very high. Thus, the threshold value for assigning a parent is set at zero, such that only parents with less than zero exclusions would be considered true parents, which leads to no parent being considered a true parent. This disadvantage disappears when the number of markers increases, as could be seen in a similar simulation with 200 markers, where their accuracy increased to 99.7%, while that of APIS was still 100.0%. While in some cases an excess of markers may be available, in many cases a “minimal” number of markers tends to be used for economic reasons, and a more powerful assignment method is required.

With APIS, the likelihood approach seemed to be more efficient than the exclusion method when the power of the marker set was limiting (typically with 48–96 markers and randomly chosen markers, both in terms of sensitivity and FDR). However, when more markers (or the “best” markers) were used, exclusion was more advantageous, with sensitivity reaching unity, very low FDR (<0.01) and excellent specificity (0.96–0.99), while the results obtained with likelihood were more variable, with higher levels of FDR (0.10 with 50% of parents genotyped) and lower specificity (≈0.90 with 90% of parents genotyped).

We showed that triploid trout should be genotyped with at least 192 SNP markers to be accurately assigned to their true parents in case some parental genotypes are missing, otherwise, 96 markers could be sufficient to get at least 95% of sensitivity with the design used. However, we recommend considering at least 192 markers because missing parental genotypes is a commonly encountered issue in breeding programs due to low DNA quality of some samples, or even lack of sampling of some parents that contributed to the mating design (Jones *et al*. 2010; Vandeputte *et al*. 2011).

The marker-centromere recombination rate was set by default to 0.5, and we showed sensitivity reached a plateau around a recombination rate of 0.5 even for populations with a highly different mean recombination rate. We did not test sets of markers with varying individual recombination rates, but these results make us confident that there should be no major impact on assignment rates in this case. Thus, the default value fixed to 0.5 in APIS for the marker-centromere recombination rate should not negatively impact the efficiency of the assignment.

The triploids used in this article were the result of a single set of chromosomes inherited from the father and a double set from the mother. But, switching mother from father in R should work in the case of triploids with 2 sets of chromosomes from their diploid father and a single one from the mother, as may happen in some plants (Wang *et al*. 2016).

In case of triploid offspring resulting from the crossing between tetraploid and diploid parents, widely used in oysters (Piferrer *et al*. 2009), the exclusion method implemented here should work with very few mistakes in case of bi-allelic markers. To do so, an “apparent” genotype should be attributed to the 4N parents in the following way: AA for AAAA, BB for BBBB, and AB for AAAB, AABB, and ABBB. Doing so, there would be only 3 cases where an existing mismatch would not be detected by the algorithm: Table 4 with 4N genotype ABBB (AB apparent) and the other parent AB or missing and Table 5 with 4N genotype AAAB (AB apparent) and the other parent AA. Thus, there should be a little loss in efficiency, with some exclusions not detected, but no exclusion of compatible parents. However, this would not work for microsatellite markers, for which, due to the high number of possible allelic variants in tetraploid parents, reduction to an apparent diploid parental genotype (required by APIS) cannot be performed.

For lines with higher levels of ploidy, like 4N trout or oysters, the method is not developed yet and will depend on the parent's ploidy. If the parents are diploids, the exclusion table will be quite simple as the sum of the 2 parents must match the offspring genotype and the likelihood table will be very similar with some differences for missing parents, as there would be no place for incertitude. Adaptation to the case of retention of polar body I or polar body II (used in mollusks) instead of polar body II only in fish would be required. For tetraploid offspring from tetraploid parents, possible outcomes are profuse and the likelihood method is more complex to quantify as we do not know yet the mode of marker segregation (double disomy, tetrasomy or a mixture of both). New tables of likelihood and exclusion would be needed, with more complexity for likelihood.

Yet, species with high ploidy levels might also be treated as diploid individuals. Following genome-wide duplication, part of the genome can be silenced or non-functionalized, and part of the genome would have a diploid behavior (Vasil’ev 2009; Nugent *et al*. 2017) or even be totally diploidized (Li *et al*. 2021). By selecting simple diploid markers from those regions, the tetraploid issue could be bypassed.

## Supplementary Material

jkae143_Supplementary_Data

## Data Availability

The APIS package is available on the CRAN at https://cran.r-project.org/package=APIS with the GNU General Public License. The data supporting the article are available at: https://doi.org/10.57745/ZHWQWQ with the Etalab Open License 2.0. Data file formats for the APIS Shiny app are specified in Supplementary Tables 2–4 in File 1. Supplemental material available at G3 online.
