# Supplementary material for: APIS: an updated parentage assignment software managing triploids induced from diploid parents
Source: G3 (Bethesda). 2024 Jul 2;14(8):jkae143. doi: 10.1093/g3journal/jkae143 (PMC11304945; doi:10.1093/g3journal/jkae143)
Supplement: jkae143_Supplementary_Data [file jkae143_supplementary_data.zip › File_S1_G3-2024-405082.docx]

Supplementary Materials

Table S1: Call rate (min, max, mean) of parent (p) and offspring (o) SNP and MAF (min, max, mean) of offspring SNP as a function of the testing phase and the number of SNP

| Dataset | Nb marker | CR  Min p | CR  Max p | CR  Mean p | CR  Min o | Cr  Max o | Cr  Mean o | Maf  Min o | Maf  Max o | Maf  Mean o |
| --- | --- | --- | --- | --- | --- | --- | --- | --- | --- | --- |
| Best | 16 | 0,98 | 1 | 1 | 1 | 1 | 1 | 0,5 | 0,5 | 0,5 |
| Best | 32 | 0,98 | 1 | 1 | 1 | 1 | 1 | 0,5 | 0,5 | 0,5 |
| Best | 48 | 0,98 | 1 | 1 | 1 | 1 | 1 | 0,49 | 0,5 | 0,5 |
| Best | 96 | 0,98 | 1 | 1 | 1 | 1 | 1 | 0,49 | 0,5 | 0,49 |
| Best | 192 | 0,97 | 1 | 1 | 1 | 1 | 1 | 0,48 | 0,5 | 0,49 |
| Best | 384 | 0,97 | 1 | 1 | 1 | 1 | 1 | 0,47 | 0,5 | 0,48 |
| Best | 500 | 0,96 | 1 | 1 | 1 | 1 | 1 | 0,46 | 0,5 | 0,48 |
| Best | 1,000 | 0,92 | 1 | 1 | 1 | 1 | 1 | 0,43 | 0,5 | 0,46 |
| Real | 16 | 0,98 | 1 | 1 | 0,98 | 1 | 0,99 | 0 | 0,45 | 0,21 |
| Real | 32 | 0,96 | 1 | 1 | 0,98 | 1 | 0,99 | 0 | 0,48 | 0,22 |
| Real | 48 | 0,91 | 1 | 1 | 0,97 | 1 | 0,99 | 0 | 0,48 | 0,2 |
| Real | 96 | 0,94 | 1 | 1 | 0,97 | 1 | 0,99 | 0 | 0,49 | 0,22 |
| Real | 192 | 0,87 | 1 | 1 | 0,97 | 1 | 0,99 | 0 | 0,49 | 0,22 |
| Real | 384 | 0,86 | 1 | 1 | 0,97 | 1 | 0,99 | 0 | 0,5 | 0,22 |
| Real | 500 | 0,81 | 1 | 1 | 0,97 | 1 | 0,99 | 0 | 0,5 | 0,22 |
| Real | 1,000 | 0,68 | 1 | 1 | 0,97 | 1 | 0,99 | 0 | 0,5 | 0,22 |
| Simu | 16 | 0,96 | 1 | 1 | 0,98 | 1 | 0,99 | 0 | 0,47 | 0,22 |
| Simu | 32 | 0,96 | 1 | 1 | 0,98 | 1 | 0,99 | 0 | 0,47 | 0,21 |
| Simu | 48 | 0,93 | 1 | 1 | 0,97 | 1 | 0,99 | 0 | 0,49 | 0,23 |
| Simu | 96 | 0,93 | 1 | 1 | 0,97 | 1 | 0,99 | 0 | 0,49 | 0,21 |
| Simu | 192 | 0,84 | 1 | 1 | 0,97 | 1 | 0,99 | 0 | 0,5 | 0,22 |
| Simu | 384 | 0,8 | 1 | 1 | 0,97 | 1 | 0,99 | 0 | 0,5 | 0,21 |
| Simu | 500 | 0,77 | 1 | 1 | 0,97 | 1 | 0,99 | 0 | 0,5 | 0,22 |
| Simu | 1,000 | 0,76 | 1 | 1 | 0,97 | 1 | 0,99 | 0 | 0,5 | 0,22 |

**Supplementary text 1: New functions of APIS beyond triploidy**

**Automated determination of the mismatch threshold for exclusion**

The first version of APIS (Griot et al., 2020) provided an automated determination of the threshold for parentage assignment to be applied to the difference (delta) between the likelihood of the first and second-best parent pairs. That first version of the software could also perform parentage assignment based on exclusion, but in this case, the user was left with the choice of the maximal number of mismatches authorized. Here we implemented an automated determination of this threshold, using a similar approach to the one pursued with the likelihood approach in the first version of APIS (Griot et al., 2020), i.e. with the aim to contain the false discovery rate (FDR) to a pre-defined level chosen by the user. From several datasets, we observed that the distribution of the number of mismatches for the second-best parent pair, in a homogeneous population, was close to being symmetrical.

Thus, we count the number individuals for which the number of mismatches of the best couple is above the median of the number of mismatches observed in the second-best couples. By multiplying this number by two, owing to the symmetry in the number of mismatches, we obtain an estimate of the number N_miss_ of individuals for which the “best” couple is in reality a “second-best” couple, meaning that the genotype of at least one of their parents is missing. If N_miss_/N , with N the total number of offspring, is below the user-chosen FDR, all offspring are assigned. If N_miss_/N>FDR, the mismatch threshold is set at a level such that the number N_2_ of second-best couples with less mismatches than that threshold satisfies N_2_/(N-N_miss_)<FDR.

**An APIS Shiny app**

To facilitate the usage of the APIS library, a shiny interface has been developed and implemented within the package via the ‘launch_shinyAPIS’ function. It consists of three phases: a formatting phase to transform genotype output files from ThermoFisher Axiom Analysis suite to a usable format for APIS, an APIS phase to perform likelihood and mismatches calculations and identify the first, second and third most likely parents for each offspring, and a verification phase to visualize the output of the assignment with summary and graphs. The shiny function can be used for diploid and triploid offspring from diploid parents and does not require advanced R skills to run.

Details on the APIS Shiny app are given below

The formatting phase requires a genotyping output file in PLINK PED (see expected format Table S2) or Variant Call Format (VCF). It also works with a .txt extension as long as the format of the file is the same as presented. This file is then transformed to match the requirement for APIS. A second file with marker names is optional, and not required to run, but if not provided, markers will be named by order of appearance. Finally, the user has to choose the type of markers between SNP (bi-allelic codominant) and microsatellites (multi-allelic codominant).

The second phase is the APIS assignment. Three files are required. The first file is the offspring genotype dataset. It can be created either manually (see the expected format Tables S3 and S4), or by the first phase in the case of diploid offspring. For triploid offspring, it can be created manually (see the expected format Table S3 and S4) or by a specific genotype clustering application for triploids (GenoTriplo R package) (Roche et al., in prep). The second file needed is the parents genotype dataset (created manually or by the first step) that can be provided by two separate files (for sires and dams) or a single one. The third file is a text file composed of at least two columns with parent names (similar to the names in the parents’ genotypes file) and their associated sex, coded 1 (or 3) for sires and 2 (or 4) for dams when a single file has been provided for parents. Then, the user can optionally provide a list of specific markers to be used for the assignment or specify a number of markers N to select. Typically, when the genotypes are obtained from an SNP chip, the number of markers available may be much higher than needed, and this option is useful to limit computation time. In that case, the best N markers, based on MAF in the offspring and call rate in the parents, will be used for the assignment. After the completion of the assignment, the user chooses the method to discriminate parental pairs (likelihood or exclusion) and the tolerated assignment error rate before saving the result.

The last (optional) phase requires 2 files: the assignment data file created by the APIS assignment phase and the mating plan of the parents. It will produce different graphs and statistics to verify the assignment made by APIS, and will verify if it fits with the expected mating plan.

Table S2: Expected format of file to run formatting phase with .ped or .txt file. A missing genotype is accounted for by “0”.

| #Sample Filename | Genotypes |
| --- | --- |
| Indiv1.CEL | T C T A G A A T C G C 0 T |
| Indiv2.CEL | T C T A G A A T C 0 C A T |
| Indiv3.CEL | T C 0 G G A A T C G C A C |
| Indiv4.CEL | T C T A G A A 0 C G C A C |
| Indiv5.CEL | T C T A G A A T C G T A T |

Table S3: Expected format of file to run APIS phase with .txt file. NA accounts for a missing genotype

| Marker1 Marker2 Marker3 Marker4 Marker5 Marker6 Marker7 Marker8 | | | | | | | | |
| --- | --- | --- | --- | --- | --- | --- | --- | --- |
| Indiv1 | A/A | A/A | NA/NA | A/G | T/T | A/C | C/C | C/C |
| Indiv2 | A/T | A/A | T/T | A/G | T/T | A/C | G/G | C/C |
| Indiv3 | A/T | A/T | T/T | A/G | T/T | A/C | G/G | C/G |
| Indiv4 | T/T | A/A | A/T | NA/NA | T/T | A/C | G/G | C/C |
| Indiv5 | A/T | A/A | A/T | A/G | T/T | A/C | G/G | C/C |

Table S4: Expected format of dataset once loaded on R to run APIS phase. NA accounts for a missing genotype

|  | **Marker1** | **Marker2** | **Marker3** | **Marker4** | **Marker5** | **Marker6** | **Marker7** | **Marker8** |
| --- | --- | --- | --- | --- | --- | --- | --- | --- |
| *Indiv1* | A/A | A/A | NA/NA | A/G | T/T | A/C | C/C | C/C |
| *Indiv2* | A/T | A/A | T/T | A/G | T/T | A/C | G/G | C/C |
| *Indiv3* | A/T | A/T | T/T | A/G | T/T | A/C | G/G | C/G |
| *Indiv4* | T/T | A/A | A/T | NA/NA | T/T | A/C | G/G | C/C |
| *Indiv5* | A/T | A/A | A/T | A/G | T/T | A/C | G/G | C/C |


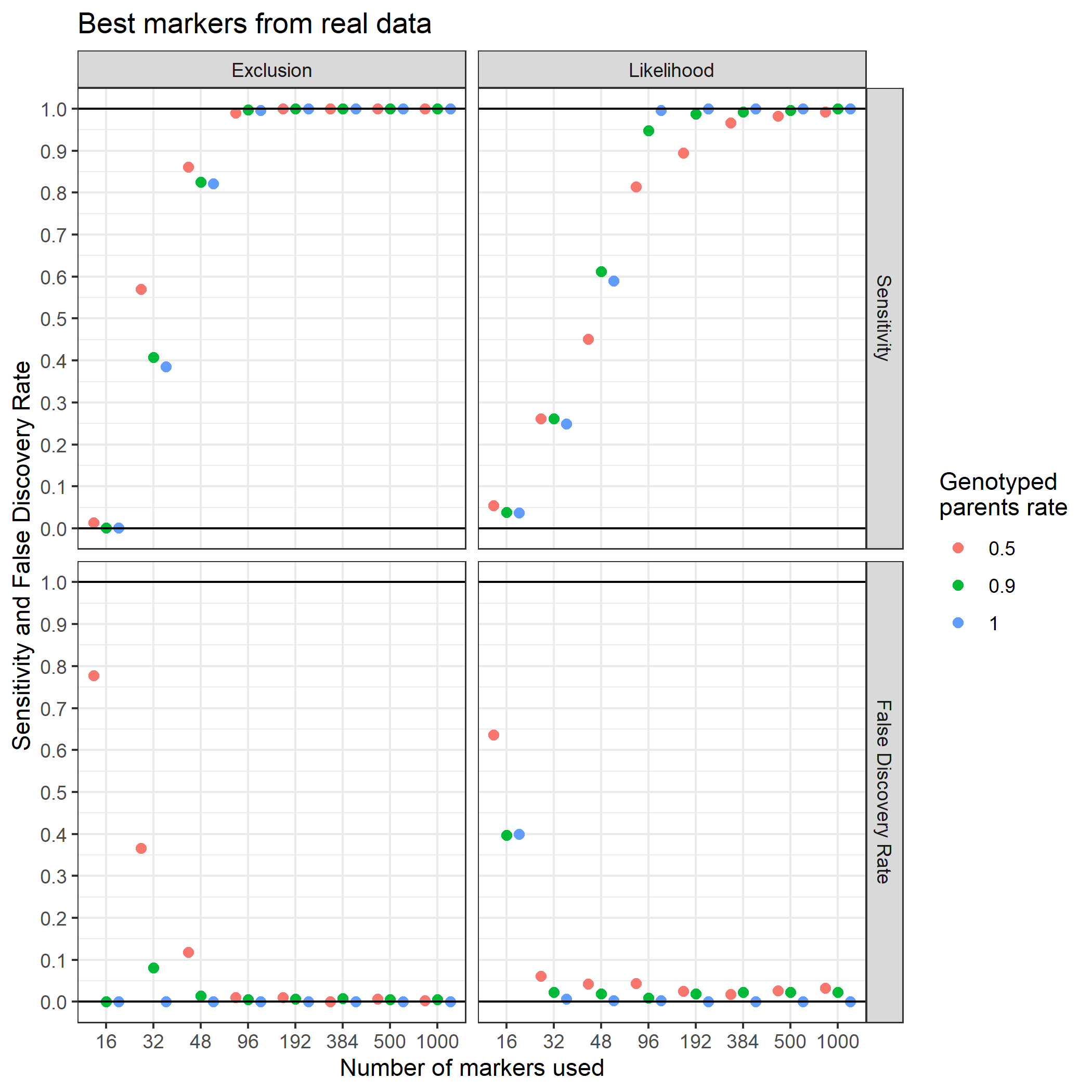


Figure S1: Sensitivity (top) and FDR (bottom) as a function of the number of SNP markers selected by CR and MAF of the offspring used for 1232 rainbow trout triploid offspring populations with 50% (red), 90% (green) and 100% (blue) of the parents genotyped with the exclusion (left) and likelihood (right) methods
